# What’s my age again? Age categories as interactive kinds

**DOI:** 10.1007/s40656-021-00388-5

**Published:** 2021-03-10

**Authors:** Hane Htut Maung

**Affiliations:** Department of Philosophy, School of Social Sciences, University of Manchester, Humanities Bridgeford Street, Manchester, M13 9PL England

**Keywords:** Age, Classification, Homeostatic Property Clusters, Interactive Kinds, Looping Effects

## Abstract

This paper addresses a philosophical problem concerning the ontological status of age classification. For various purposes, people are commonly classified into categories such as “young adulthood”, “middle adulthood”, and “older adulthood”, which are defined chronologically. These age categories *prima facie* seem to qualify as natural kinds under a homeostatic property cluster account of natural kindhood, insofar as they capture certain biological, psychological, and social properties of people that tend to cluster together due to causal processes. However, this is challenged by the observation that age categories are historically unstable. The properties that age categories are supposed to capture are affected by healthcare and cultural developments, such that people are staying biologically, psychologically, and socially young for longer. Furthermore, the act of classifying people into age categories can bring about changes in their behaviors, which in turn alter the biological, psychological, and social properties that the categories are supposed to capture. Accordingly, I propose that age categories are best understood as interactive kinds that are influenced in dynamic ways by looping effects. I consider some implications of these looping effects for our classificatory practices concerning age, including how different disciplines may need to review the ways they define and use age categories in their inductive inferences.

## Introduction

In our everyday interactions and in public policy, we commonly use chronologically defined age categories such as “young adulthood”, “middle adulthood”, and “older adulthood” to classify ourselves and one another, make inferences about groups of people, and inform our social practices. This paper offers a philosophical analysis of the ontological status of age classification. In §2, I explore the possibility of taking age categories to be natural kinds. I consider a homeostatic property cluster account of natural kindhood (Boyd, 1999a), which *prima facie* seems to accommodate many features of age categories. This view, however, is confounded by the observation that age categories are affected by dynamics that make them historically unstable. In §3, I examine the ways in which healthcare and cultural developments are changing some of the properties that age categories are supposed to capture, such that people are staying biologically, psychologically, and socially younger for longer. This does not necessarily undermine the claim that age properties refer to homeostatic property clusters, but shows that these clusters are, to certain extents, historically and geographically contingent. Nonetheless, there remains a further challenge. In §4, I examine the ways in which the very act of classifying people into age categories brings about changes in their behaviors, which in turn alter the properties of people that age categories are supposed to capture. Accordingly, I propose that age categories are best understood as interactive kinds that are influenced in dynamic ways by looping effects (Hacking, 1995a). In §5, I consider the implications of this philosophical analysis for our classificatory practices concerning age, including the extents to which the looping effects confound the uses of age categories by different disciplines and how these disciplines might need to review the ways they approach age classification.

## Age categories and homeostatic property clusters

Classification serves a range of useful epistemic and practical purposes. Where categories capture stable similarities within a group and differences between groups, they can be used to support inductive inferences. In turn, these inductive inferences can inform interventions. For example, members of a given species have similar developmental histories and can be expected to require similar diets, samples of a given compound share the same molecular structure and can be expected to react in similar ways to a reagent, and cases of a given infectious disease have similar etiological agents and can be expected to respond to similar treatments.

Philosophers often refer to these theoretically significant and robustly generalizable categories as natural kinds. Precisely what constitutes natural kindhood is a contested topic and different philosophers have suggested different criteria. The essentialist account suggests that members of a natural kind share the same intrinsic essence, such as samples of water all sharing the molecular structure H_2_O (Ellis, 2001). The etiological account suggests that members of a natural kind share the same causal history, such as members of the species *Canis lupus* being descended from a common evolutionary ancestor (Millikan, 1999). The homeostatic property cluster account suggests that members of a natural kind tend to share a cluster of properties that are contingently connected by causal mechanisms, such as members of the species *Canis lupus* tending to have long snouts, strong canines, and tails (Boyd, 1999a). However, despite these diverging accounts, philosophers generally agree that members of a natural kind are alike in virtue of certain properties that are generalizable enough to support our epistemic activities. Rachel Cooper notes that “natural kinds are often thought to appear in natural laws, to function in explanations, and to support inductive inferences” (Cooper, 2004, p. 84). Likewise, Muhammad Khalidi endorses “a weak realist view that considers as real any kind that plays an indispensable role in explaining phenomena, making successful predictions, and otherwise featuring in successful inductive inference” (Khalidi, 2010, p. 358). Accordingly, because our classificatory activities are closely tied to our inferential purposes, an epistemic approach to natural kindhood acknowledges that what we take to be a natural kind is partly dependent on our conventions, practices, and interests (Dupré, 1993).

A classification we commonly apply to ourselves and one another in everyday discourse is age. We frequently characterize ourselves and one another as “young”, “middle aged”, and “old”. These categories have descriptive roles, such as “infancy” and “early childhood” being used to describe certain developmental stages. However, some of the categories, such as “young adult” and “older adult”, also have normative roles, as they are considered to connote certain expected ways of behaving, as typified by the colloquial expression “act your age” (Settersten and Mayer, 1997). Indeed, the title of this paper is inspired by the song “What’s My Age Again?” by the punk rock band Blink-182, which revolves around a man’s failure to behave in a manner that is deemed appropriately mature. In more formal contexts, demographers, public health organizations, and policy makers frequently use categories such as “young adulthood”, “middle adulthood”, and “older adulthood” to inform research, practice, and policy. The diverse uses have included, among other things, measuring suicide rates in different age cohorts (Suicide Prevention Resource Center, 2020), studying differences in voting patterns between young people and older people (Prosser et al., 2018), implementing health interventions in different age groups (Mortensen & Falk, 2018), and deciding which members of society are eligible for senior social programs (Bunis, 2020).

Typically, these age categories are defined chronologically, such that a person is considered “young” if he or she is below a certain chronological age, “middle aged” if he or she falls within a certain chronological age range, and “old” if he or she is above a certain chronological age. Elizabeth Lindemann and colleagues suggest the following classification, which uses the National Institute of Child Health and Human Development guideline for the pediatric categories and the United States Census guideline for the adult categories:

Infant and Toddler (birth to 24 months of age), Early Childhood (2–5 years), Middle Childhood (6–11 years), and Early Adolescence (12–18 years) … Young Adulthood (19–44 years), Middle Adulthood (45–64 years), and Older Adulthood (65 years and older). (Lindemann et al., 2017, p. 1170)

Although this formal classification system is widely used, it is by no means universally accepted, as the precise boundaries of “young adulthood”, “middle adulthood”, and “older adulthood” are contested. As we shall later see, these contested boundaries reflect the unstable characteristics of some of the categories and the varying classificatory interests of different disciplines.

Historically, the use of chronological age to define age categories was partly motivated by various political and economic interests. With the development of industrialization in the eighteenth century and the establishment of formal education, there was an incentive to structure society in a way that sets out the points of entry and exit from the labor market (Neugarten, 1981). Governments began to record birth dates accurately and to use chronological age to organize cohorts for schooling, employment, and retirement. Accordingly, the popular notion that 65 marks the beginning of “older adulthood” is partly attributable to fact that for some time this was the age at which a person would customarily begin to receive his or her pension (Salter, 2003).

Although some age categories were constructed for political and economic purposes, they are not arbitrary groupings, but are supposed to capture genuine properties of people. These properties roughly correspond to what James Birren and Walter Cunningham (1985) call biological age, psychological age, and social age. Biological age is defined by a person’s “present position with respect to his potential life span” and is measured by the “assessment of functional capacities of vital or life-limiting organ systems”. Psychological age is defined by the “behavioral capacities of individuals to adapt to changing demands”, including the uses of “memory, learning, intelligence, skills, feelings, motivations, and emotion for exercising behavioral control or self-regulation”. Social age is defined by the person’s “roles and habits with respect to other members of the society of which he is a part. An individual may be older or younger depending on the extent to which he shows the age-graded behavior expected of him by his particular society or culture” (Birren & Cunningham, 1985, p. 8).

These three characteristics can come apart and, as we shall see, the ways they relate to chronological age are not straightforward (Nathan, 2021). Nonetheless, the characteristics do tend to be contingently correlated with one another by means of causal processes. Plausibly, biological age is often correlated with psychological age, because the physiological capacities of vital organ systems, such as cardiovascular, metabolic, and nervous systems, can set constraints on a person’s ability to utilize certain cognitive capacities, including memory, learning, and emotional regulation. For example, as a group, older adults tend to have lower resting metabolic rates (Jazwinski & Kim, 2019), lower cerebrovascular blood flows (Izzo et al., 2018), and higher concentrations of neural protein aggregates (Del Tradici & Braak, 2008) than young adults. These, along with other biological characteristics, are suggested to influence capacities such as memory, reasoning, spatial visualization, and processing speed (Salthouse, 2009).

How social age is related to biological age and psychological age is more complicated, because the roles and habits expected at a given age are contingent on the norms and values of the society wherein the person is situated. Accordingly, the characteristics that are used as indicators of social age are likely to vary across cultures. However, within a given cultural context, social age may be correlated with biological age and psychological age, insofar as people’s behavioral dispositions and assigned roles may be influenced by judgments of their physiological and cognitive capacities. For example, Nir Enyon and colleagues note that “muscle strength and mass decline 30–50% between the ages of 30 and 80” and that the “declining strength reduces the capacity to carry out basic activities of daily life and puts people at risk for falls and dependence on others” (Enyon et al., 2009, p. 55). As such, older adults may be exempted from work and considered eligible for senior social programs, because they are considered to have different physiological capacities and healthcare requirements from young adults. Biological senescence has also been associated with decreasing fecundity and increasing mortality, and so different biological ages tend to be correlated with different social roles with respect to reproduction and childrearing (Giaimo, forthcoming).

Biological age, psychological age, and social age are commonly assumed to correlate with chronological age, because the effects of the various factors that occasion the respective physiological, cognitive, and behavioral changes tend to accumulate over chronological time. At the macroscopic level, various social and environmental factors, including deprivation, food poverty, carcinogen exposure, and stress, have been shown to be causal factors that have cumulative effects on people’s physiological and cognitive capacities (Walker, 2018). At the microscopic level, the cumulative effects of these social and environmental factors, in conjunction with genetic risk factors, influence the rates at which the cellular processes associated with senescence occur (Adams & White, 2004). And so, chronologically defined age categories are used as rough indicators of certain physiological, cognitive, and behavioral properties of people, insofar as biological age, psychological age, and social age are assumed to correlate roughly with chronological age. These correlations are what underpin their being used to make inferences about people’s healthcare requirements, behaviors, and capacities to assume certain social roles (Chudacoff, 1989).

In light of the above, age categories do seem to have some features associated with natural kinds. However, they are not natural kinds according to an essentialistic account, as the members of a given age category do not all share an intrinsic essence that is not instantiated by members of other age categories. Rather, they are closer to what Richard Boyd (1999a) calls homeostatic property clusters. A homeostatic property cluster is a set of properties that tend to cluster together due to contingent causal processes called homeostatic causal mechanisms. These homeostatic causal mechanisms are probabilistic rather than deterministic, such that the presence of one property does not necessitate the presence of another property, but makes it statistically more likely. Under this account, a category is a natural kind if its members share a sufficient number of properties. Importantly, the members may instantiate different combinations of properties and there is no single property that is essential for membership of the kind. For example, members of the species *Canis lupus* tend to have long snouts, strong canines, and tails due to their having similar genetic ancestries and similar developmental environments, but English Bulldogs have short snouts and Pembroke Welsh Corgis lack tails.

Likewise, age categories could be considered to correspond to clusters of biological, psychological, and social characteristics that tend to correlate with one another due to contingent causal processes. For example, given that members of the category “older adulthood” have endured the cumulative effects of biological and environmental stressors for greater lengths of time than members of the category “young adulthood”, older adults are more likely to share certain physiological, cognitive, and behavioral properties, which they do not share to the same degrees with young adults. Of course, there are significant variations among members of the category and not all members of the category instantiate the same combinations of properties. For example, the markers of biological age have been shown to vary between different people of the same chronological age and also between different cells within the same organism (Nathan, 2021). Nonetheless, despite these variations, it is plausible that homeostatic causal mechanisms can occasion some rough differences between “young adulthood”, “middle adulthood”, and “older adulthood” that are statistically relevant.

It is important to note that not all of the properties in the cluster are intrinsic properties of individuals. Some properties may obtain due to the stabilizing influences of conditions that are externally located. As Crawford Elder notes, “members of some natural kinds may reliably present the same distinctive packet of observable properties … because of the moulding of a common environment” (Elder, 1995, p. 516). Boyd uses the example of a meteorological kind and notes the “kind of stability which defines a natural kind of storm system, for example, may depend, unexpectedly perhaps, on the nature of weather systems distant from the storm itself” (Boyd, 1999b, p. 84). The influences of extrinsic properties on age categories are especially obvious with respect to social age, as people’s social behaviors and assigned roles are shaped by the norms and values of society. However, to some extents, the roles of extrinsic properties also apply to biological age and psychological age, insofar as the effects of social and environmental stressors can set constraints on people’s physiological and cognitive capacities (Walker, 2018).

Based solely on what has been discussed so far, there appears to be little issue with construing age categories as homeostatic property clusters. A given age category, such as “older adulthood”, roughly corresponds to a set of biological, psychological, and social characteristics which tend to cluster together due to causal processes. These causal processes include the cumulative effects of biological and environmental stressors over chronological time, the constraints that physiological capacities can set on cognitive capacities, and the interactions between these capacities and external influences in the social environment. However, this view is confounded by the observation that the physiological, cognitive, and behavioral properties that age categories are supposed to capture are affected by dynamics in ways that make them historically unstable. In the following section, I consider how healthcare and cultural developments are changing the characteristics of people, such that they are staying biologically, psychologically, and socially young for longer.

## Age categories are historically and geographically contingent

We have seen that chronologically defined age categories serve to classify people into groups based on certain shared characteristics of their respective members. However, insofar as chronological age is a continuous variable, these age categories do not correspond to discrete kinds demarcated by “zones of rarity” (Kendell & Jablensky, 2003), but to regions on a continuous distribution of properties that shade into one another. As with any partitioning of a continuous distribution into categories, the precise boundaries between the categories must be chosen conventionally or at least informed by our values and interests. In the previous section, I noted that the boundaries between “young adulthood”, “middle adulthood”, and “older adulthood” assumed by Lindemann and colleagues are based on the age ranges used in the United States Census guideline (Lindemann et al., 2017). These, in turn, are informed by considerations regarding “our familial, educational, and occupational institutions”, as well as by judgments regarding people’s healthcare requirements and capacities to perform certain roles (Settersten & Mayer, 1997, p. 235).

However, since these chronological boundaries between the age categories were initially established, healthcare and cultural developments have been changing the characteristics of people in ways that are allowing them to stay biologically, psychologically, and socially younger for longer. Here, I am using the term healthcare broadly to encompass developments in health education, resource distribution, illness prevention, and treatment of disease. For example, in the United States, the prevalence of tobacco smoking has decreased dramatically over the past four decades, with an associated improvement in population health (Wang & Preston, 2009). Therapeutic interventions and preventative measures to manage blood pressure and cholesterol have also become more widely implemented over the past four decades. Given that hypertension and hypercholesterolemia are risk factors that impair cardiovascular, cerebrovascular, and metabolic capacities, such measures are plausibly associated with an improvement in population health (Hajjar & Kotchen, 2003).

Partly due to these and other developments, average life expectancies have been increasing. In the United Kingdom, the average life expectancies for men and women were respectively 85 and 87 in 2017, whereas they were respectively 77 and 82 in 1981 (Office for National Statistics, 2019). The rates of senescence have also been decreasing. In a study by Morgan Levine and Eileen Crimmins (2018), people in a sample from 1988 to 1994 were matched according to their chronological ages with people in a sample from 2007 to 2010 and their respective physiological characteristics were compared. Various metabolic, cardiovascular, respiratory, renal, hepatic, and inflammatory parameters were recorded, including “glycosylated hemoglobin, total cholesterol, systolic blood pressure (BP), ratio of forced expiratory volume at 1 s (FEV1) to forced vital capacity, serum creatinine, serum alkaline phosphatase, serum albumin, and C-reactive protein (CRP)” (Levine & Crimmins, 2018, p. 390). These were entered into an algorithm to calculate the biological ages of the people, which in turn allowed comparisons of the biological ages of the people from the two different historical periods relative to their chronological ages. The results indicated that the people in the sample from 2007 to 2010 had significantly lower biological ages relative to their chronological ages than the people in the sample from 1988 to 1994. That is to say, the rates of senescence had decreased over the two decades that were studied, such that people are staying biologically younger for longer relative to their chronological ages.

Further to these healthcare developments, there have been cultural developments associated with changes in people’s social ages relative to their chronological ages. Compared to several decades ago, people are continuing their educations for longer, finding partners later, and are having children later. Accordingly, Susan Sawyer and colleagues have proposed raising the chronological boundary between “adolescence” and “young adulthood” from age 19 to age 24 (Sawyer et al., 2018). People are also remaining employed for longer and are retiring later. For example, in the United Kingdom, politicians have advocated a gradual increase in the state pension age from 65 to 70 (Salter, 2003). And so, the social roles and behavioral capacities expected at various chronological ages are changing in such ways that people are generally staying socially younger for longer.

Given the changing characteristics of people across history relative to their chronological ages, there have been calls to revise the chronological age boundaries that define our age categories, in order that these categories can continue to capture the same biological, psychological, and social properties that they had previously captured. As noted above, “adolescence” has been suggested to finish at 24 rather than at 19 (Sawyer et al., 2018). In addition, the age at which “older adulthood” begins is being reviewed. For example, the Joint Committee of the Japan Gerontological Society and the Japan Geriatrics Society have proposed that “older adulthood” begins at 75 rather than at 65 (Ouchi et al., 2017). The boundaries of “young adulthood” and “middle adulthood” could also be considered to be shifting upwards, as people are staying biologically younger for longer, are remaining active for longer, are finding partners later, and are having children later. Hence, at least one political commentator has proposed that “young adulthood” extends beyond one’s early 40 s (Bush, 2018), while a survey has suggested that most people in the United Kingdom think “middle adulthood” begins at 48 (Smith, 2018).

The historical contingency of age classification is further compounded by its geographical contingency. Given that different parts of the world vary with respect to their public health systems, social conditions, and levels of poverty, the rates of senescence also vary across populations. Using a combination of biological and social measures, the World Health Organization (2002) has suggested 50 as the chronological age at which “older adulthood” begins in the parts of Africa most affected by poverty, compared to 65 in Europe and North America.

The changing characteristics of people that result from the aforementioned healthcare and cultural developments present a practical challenge to age classification, because they suggest that the properties of people that are being captured by chronological age categories are not stable, but are historically and geographically contingent. However, I argue that this on its own does not necessarily undermine the view that age categories are homeostatic property clusters. Many other things that we generally consider to be natural kinds are also to varying extents historically and geographically contingent, such as biological species and some disease kinds. For example, reticulated pythons of the species *Malayopython reticulatus* exhibit considerable differences with respect to their sizes and appearances across geographical regions, partly due to genetic variations and partly due to the effects of different environmental conditions. The species is generally recognized as the longest species of snake on the world, although only populations on mainland Southeast Asia tend to reach such large sizes, while some of the populations on the islands of Indonesia, Malaysia, and the Philippines tend to be much smaller (Auliya et al., 2002). These geographical differences reflect changes in the morphological characteristics of reticulated pythons across history as they diversified into new habitats. Nonetheless, despite these historical and geographical changes, the species *Malayopython reticulatus* can be considered a homeostatic property cluster, insofar as the members of the species tend to share various biological properties that cluster together due to robust causal processes, such as their similar genetic ancestries and similar developmental histories. While some of the morphological characteristics associated with the species have changed and diversified, the category still tracks a fairly robust cluster of biological properties.

Another example of something that is an inductively useful category yet is to some extent historically contingent is the disease type II diabetes mellitus. This is plausibly a homeostatic property cluster, as the diagnostic category picks out a cluster of metabolic properties, including insulin resistance and glucose intolerance, which are causally related. However, due to various healthcare and cultural developments, the demographic and clinical characteristics associated with type II diabetes mellitus have been changing. While the disease used to affect predominantly older adults, it is now also diagnosed in young adults, adolescents, and children, due to the increasing rates of obesity in these populations. Accordingly, it is no longer characterized as “adult-onset diabetes”. Furthermore, the increasing number of cases of type II diabetes mellitus that are related to obesity has changed it from a disease that was largely controllable through lifestyle changes and oral antidiabetic drugs to a disease that often involves more intensive therapies to reduce the risks of comorbidities (Brunton, 2008). Again, despite these changes in some of the characteristics associated with the disease, it is generally unproblematic to consider type II diabetes mellitus a homeostatic property cluster, insofar as cases of the disease tend to share a cluster of metabolic properties that are causally related.

Similarly, based on what has been discussed so far, age categories such as “adolescence”, “young adulthood”, and “older adulthood” could still be taken to pick out clusters of causally related physiological, cognitive, and behavioral properties, even though healthcare and cultural developments are changing the chronological ages at which these clusters tend to obtain. Just as our descriptions of the demographic groups who can develop type II diabetes mellitus must be revised in order for the diagnostic category to capture the increasing range of people who exhibit the relevant metabolic characteristics, the chronological age boundaries that are used to define age categories must be revised in order for these categories to capture the equivalent clusters of physiological, cognitive, and behavioral properties that the categories had previously captured. In other words, categories such as “young adulthood” and “older adulthood” can still correspond to homeostatic property clusters, but the chronological age boundaries that demarcate these categories have to be raised in order to reflect the ways in which the respective homeostatic property clusters are obtaining at higher chronological ages.

And so, we have seen how healthcare and cultural developments have been altering the characteristics of the members of age categories. Such historical contingency and geographical contingency do not necessarily undermine the claim that age categories are homeostatic property clusters, although they do indicate that the chronological age boundaries that are used to define these categories must continue to be reviewed in light of changing healthcare and cultural contexts. This is an issue that is recognized by researchers, practitioners, and policy makers, who have been developing proposals to revise these chronological age boundaries accordingly. However, I argue that age categories are affected by a further set of processes that present more trouble for age classification. In addition to the effects of people’s changing health behaviors on the characteristics associated with age categories, the age categories in turn have effects that change people’s health behaviors. Hence, the interactions are not unidirectional, but involve bidirectional feedback. As we shall see, such feedback is partly due to the ways in which the members of these categories are motivated to alter the ways in which they are classified. In light of these reciprocal interactions between our classificatory practices and the people being classified, I suggest that age categories are best understood as interactive kinds that are influenced in dynamic ways by looping effects.

## Age categories as interactive kinds

In the philosophical debate about natural kindhood, Ian Hacking (1995a) describes a process he calls “the looping effects of human kinds”. This has been articulated in various ways by Hacking. In *Rewriting the Soul*, he writes:

We tend to behave in ways that are expected of us, especially by authority figures—doctors, for example … People classified in a certain way tend to conform to or grow into the ways that they are described; but they also evolve in their own ways, so that the classifications and descriptions have to be constantly revised. (Hacking, 1995b, p. 21)

Later, in *The Social Construction of What?*, he writes:

We are especially concerned with classifications that, when known by people or those around them, and put to work in institutions, change the ways in which individuals experience themselves—and may even lead people to evolve their feelings and behavior in part because they are so classified. (Hacking, 1999, p. 104)

Hacking’s central claim is that for at least some properties, classifying people into categories based on these properties can bring about changes in the people’s behaviors, which in turn alter the properties of the people that the categories are supposed to capture and the wider social attitudes towards these people.

The looping effects are reciprocal. One direction involves the influences of the categories on the people being classified. The other direction involves the influences of the people being classified on the categories. Hacking (1995b) illustrates this with the example of multiple personality disorder. The diagnostic category, multiple personality disorder, had previously been used since the 1840s to classify psychiatric patients who seemed to exhibit multiple enduring personality states. Each patient would usually have exhibited two personality states that were dissociated from each other. However, when patients exhibiting greater numbers and varieties of personality states began to appear on television shows and in magazines in the 1980s, more and more patients diagnosed with multiple personality disorder began to present with similar symptoms. Consequently, the characteristics of the people associated with the category multiple personality disorder changed from the 1840s to the 1980s due to the looping effects of classifying people on their behaviors.

Hacking refers to the kinds that are affected by looping effects as interactive kinds. These are contrasted with what he calls indifferent kinds, which are not affected by such looping effects. Examples of indifferent kinds are paradigmatic natural kinds, such as quarks, electrons, plutonium, and sulphur. As Hacking notes, the “classification ‘quark’ is indifferent in the sense that calling a quark a quark makes no difference to the quark” (Hacking, 1999, p. 105). By contrast, interactive kinds are usually involved when classifying people, who are aware of the terms that are applied to them. As Cooper (2004) notes, these terms often elicit value judgments, which motivate people to attempt to alter the ways in which they are classified. For example, the derogatory and discriminatory social attitudes against people classified as “obese” not only negatively affect the thoughts and behaviors of people who get classified as “obese”, but also make others want to avoid being classified as “obese” (Cooper, 2004, p. 78).

Age categories such as “young”, “middle aged”, and “old” also elicit normative judgments. In much of Europe and North America, social attitudes tend to be negative towards old age and positive towards youth. As Johanna Goll and colleagues note, “[s]ocietal discourses commonly associate youthfulness with valued traits such as independence, economic productivity and usefulness, and ageing with characteristics deemed intensely negative, like dependency and uselessness” (Goll et al., 2015, p. 13). This reflects a common metaphor in the evolutionary literature on ageing, which values the reproductive virility associated with youth and frames senescence as decline (Garson, forthcoming). Such valorization of youth intersects with patriarchy and capitalism to drive a vast industry that promotes efforts to stay young and delay senescence (Richeson & Shelton, 2006, p. 174). Furthermore, some aspects of senescence are also being brought within the domain of medicine. Arthur Caplan writes:

Pathological change is inevitably defined as constituting any morbid process in the body. And morbid processes are usually defined in terms of disease states of the body. Regardless of the circularity of this concept, ageing would therefore seem to have a *prima facie* claim to being counted as a disease. (Caplan, 2005, p. 73)

This is not to say that medical professionals actually consider senescence to be a disease, but that the notions of ageing and health have become very closely linked (Sholl, forthcoming). Accordingly, many healthcare and lifestyle interventions target various processes associated with senescence. For example, the National Health Service in the United Kingdom has a tool to “check your heart age”, which includes information about how diet, exercise, and antihypertensive treatment can maintain a young biological age relative to one’s chronological age (National Health Service, 2016).

Given society’s valorization of youth, stigmatization of old age, and promotion of activity that delays senescence, the practice of classifying people as “young”, “middle aged”, and “old” motivates people to attempt to alter the ways in which they are classified. People take measures to avoid being classified as “old” and to continue being classified as “young”, as well as to change the characteristics associated with their chronological age cohorts. This is especially visible with respect to social age. In a recent qualitative study by Goll and colleagues examining the attitudes and behaviors of people aged over 60, many participants were reported to have “made frequent attempts to distance themselves from ‘old’ people, often describing themselves as youthful and ‘young at heart’” (Goll et al., 2015, p. 11). And so, people are rejecting the expected behaviors and social roles associated with “older adulthood” for longer.

This trend also applies to the category of “middle adulthood”. The internet contains many popular magazine articles such as “27 Celebrities Who Look Nothing Like Their Actual Age” (Chandra, 2020) and “These 20 Celebrities Just Don’t Seem to Age” (Freedman, 2020), which are intended to show that famous people are appearing and behaving younger for longer relative to their chronological ages. Another popular media theme is “40 is the New 30” (McLaren, 2001), which suggests that people are flouting the expected behaviors, social roles, and appearances associated with “middle adulthood”, so that the period of “young adulthood” is being extended. That is to say, people are socially remaining in “young adulthood” for longer due to the effects of their attitudes towards how they are classified.

In addition to the influences on social age, looping effects are also exerting influences on biological age and psychological age. In a study by Namkee Choi and colleagues examining the discrepancies between chronological age and felt age, older adults were reported to have “more reasons and greater motivation for feeling younger than their chronological age as a self-enhancement or self-protection strategy” than younger adults (Choi et al., 2014, p. 461). Interestingly, the participants aged between 65 and 79 who were most motivated to feel, look, and act younger exhibited fewer indicators of biological and psychological senescence, as indicated by fewer health problems, fewer cognitive concerns, and fewer impairments in daily activities. This suggests that the normative connotations associated with the categories “old” and “young” are motivating people to modify their behaviors in ways that slow the rates of biological and psychological senescence, which in turn alter how they are classified.

The looping effects described herein further compound the effects of healthcare and cultural developments on the rates of senescence that were discussed in the previous section (Levine & Crimmins, 2018). Not only are the biological, psychological, and social characteristics that age categories are supposed to capture historically and geographically contingent, but they are also reflexive. That is to say, the chronological age boundaries that are used to define the age categories are not only shifting due to changes in the healthcare and cultural environments, but also due to the effects of the classificatory practices themselves. People are staying biologically, psychologically, and socially younger, partly because the desirability of being classified as “young” motivates people to act in ways that allow them to stay biologically, psychologically, and socially younger.

At this point, some concessions must be made. To suggest that age categories are influenced by looping effects is not to suggest that people are in complete control of how quickly or slowly they age. People’s health behaviors can, influence the rates at which they age biologically and psychologically, but there are obviously limits to what people can achieve. Given our current medical, technological, and environmental constraints, the measures people can take to delay senescence are far exceeded by the effects of the various processes that cause senescence. Hence, in the aforementioned study by Choi and colleagues, being motivated to feel, look, and act younger was associated with fewer indicators of biological and psychological senescence in participants aged between 65 and 79, but not in participants aged over 80 (Choi et al., 2014).

Furthermore, I am not suggesting that all age categories are amenable to being influenced by looping effects to the same degrees. For example, “infancy” and “early childhood” are influenced to much lesser degrees by looping effects than “young adulthood”, “middle adulthood”, and “older adulthood”, because children are much less concerned than adults by the categories assigned to them. This is not to say that “infancy” and “early childhood” are indifferent kinds. Jaakko Kuorikoski and Samuli Pöyhönen (2012) argue that looping effects can act on people even if the people are not concerned by the categories assigned to them. They describe a process called motivation crowding out, where social institutions conceptualize people in certain ways and formulate incentives that implicitly steer people to act in accordance with these conceptualizations. For example, conceptualizing “infancy” and “early childhood” as states of innocence and incapacity can incentivize caregivers to act in certain ways towards and provide certain environments for children, which influence how the children in these categories behave, grow, and develop, even though the children themselves may not be explicitly concerned by how they are conceptualized.

The categories of “young adulthood”, “middle adulthood”, and “older adulthood” may be influenced by certain forms of motivation crowding out as well. However, they are also influenced by further sets of looping effects due to the ways in which adults are concerned by the categories assigned to them. Given these additional looping effects, these age categories are more unstable than “infancy” and “early childhood”. Moreover, while the boundaries of “infancy” and “early childhood” are largely informed by biological and developmental considerations, the boundaries of “young adulthood” and “middle adulthood” are to greater extents informed by social and cultural considerations, including conventions concerning expected roles and behaviors (Smith, 2018). Accordingly, the boundaries of “young adulthood” and “middle adulthood” are more strongly influenced by cultural feedback from the changes in public attitudes and social roles brought about by our classificatory practices.

The dynamics that influence the category of “adolescence” are slightly more complex. Of course, an important property that characterizes this age category is puberty, which is the developmental process during which sexual maturity is reached. The average age of the onset of puberty is usually taken to define the beginning of “adolescence”. However, puberty is neither necessary nor sufficient for membership of this age category. First, the concept of “adolescence” is not only informed by biological and developmental considerations, but also by normative considerations regarding expected behaviors and social roles, which are historically and geographically contingent. For example, in the United States, “adolescence” was not formulated as a relevant age category until secondary education became widespread (Kett, 2003). Second, while “adolescence” often coincides with the age of puberty, a person who has not commenced puberty, perhaps due to androgen insensitivity or the use of hormonal medication, but nonetheless performs the expected social role would still be classified in category “adolescence” if he or she falls within the relevant chronological age range. Given this complexity, the boundaries and characteristics of “adolescence” can be influenced by different sets of dynamics. In one direction, due to changing population health and nutrition, the average age of onset of puberty has been decreasing over the past century, which has lowered the chronological age that “adolescence” is considered to begin (Pierce & Hardy, 2012). In the other direction, the characteristic behaviors and social roles of people have been changing, such that they are continuing their educations for longer, living with their parents for longer, and beginning their careers later. Accordingly, as noted above, scholars have proposed raising the chronological boundary between “adolescence” and “young adulthood” from age 19 to age 24 (Sawyer et al., 2018).

To summarize so far, chronologically defined age categories are best understood as interactive kinds whose characteristics and boundaries are influenced by looping effects. However, such looping effects influence different age categories in different ways and to different extents. Accordingly, some age categories are more dynamic and unstable than others. The categories of “infancy” and “early childhood” are relatively stable clusters that are largely informed by developmental characteristics, although these can still be altered to certain extents by looping effects. By contrast, the categories of “young adulthood” and “middle adulthood” are more conventional categories that are more readily altered by people’s attitudes, and so are significantly more unstable. The categories of “adolescence” and “older adulthood” are partly informed by puberty and senescence respectively, which can be altered to certain extents by biological feedback, but they are also partly informed by expected behaviors and roles, which can be altered to greater extents by cultural feedback. In the following section, I consider the implications of this analysis for age classification, including whether it undermines the view that age categories are natural kinds and whether it requires us to revise our classificatory practices.

## Revising our classificatory practices

We have seen that age categories are interactive kinds influenced by looping effects. To recapitulate, interactive kinds are kinds whose members are significantly modified by the ways they are classified into categories, which in turn can lead to those categories themselves being modified. In his earlier work on natural kindhood, Hacking (1995a) suggests that interactive kinds cannot be natural kinds. Herein, I examine why he thinks this is so and apply it to the topic of age classification. Ultimately, I propose that whether or not age categories are natural kinds cannot be determined a priori from his argument, but depend on the specific interests and purposes of different disciplines.

A reason why interactive kinds might not be considered natural kinds is that the looping effects make them unstable categories, thus confounding our attempts to use them to support inductive inferences. However, this reason on its own seems insufficient. As Khalidi (2010) argues, looping effects influence a much wider range of categories than Hacking suggests, including many categories we paradigmatically consider to be natural kinds. Accordingly, many of the natural kinds that Hacking assumes are indifferent kinds are actually interactive kinds. James Bogen (1988) considers the looping effects that influence corn and marijuana. The classification of corn as a crop was associated with the selective breeding and intensive farming of corn plants, which in turn led to the characteristics of corn plants changing across generations. Despite this, the corn species *Zea mays* is still generally considered a natural kind. Similarly, the classification of marijuana as a drug was associated with the intensive growing of marijuana plants using artificial lighting, which in turn led to the characteristics of marijuana plants changing across generations. Again, despite this, the marijuana species *Cannabis sativa* is still generally considered a natural kind.

However, Cooper (2004) notes that there is a difference between how feedback works on marijuana plants and how feedback works on people. Classifying people as “young” or “old” alters people’s characteristics because people care about how they are classified, and so are motivated to change their behaviors to change how they are classified. By contrast, while classifying marijuana as a drug does alter the characteristics of marijuana plants across generations, this happens because the classification leads to changes in how the marijuana plants are grown, not because marijuana plants are motivated to change their characteristics. And so, Hacking’s claim that interactive kinds are not natural kinds does not rest on the mere fact that feedback occurs, but rather on the fact that people care about how they are classified.

This raises the question of why people’s caring about how they are classified should preclude the categories from being natural kinds. A potential reason is that it suggests that the classification is not impartial, but is dependent on people’s attitudes. However, as noted by Cooper (2004), there are strong and weak senses in which categories can be dependent on people’s attitudes. In the strong sense, the changing classification is entirely due to changes in social attitudes. For example, social attitudes regarding what clothes qualify as “fashionable” have changed across history, such that an outfit that was considered “fashionable” a century ago may not be considered “fashionable” today. In this case, the way the outfit is classified has changed because the norms and standards that inform judgments about what is “fashionable” have changed, but the characteristics of the outfit have not themselves changed. In the weak sense, the changing classification is influenced by people’s attitudes, but involves changes in the characteristics of what is being classified. For example, a person diagnosed as “obese” may try to lose weight so that he or she is no longer classified as “obese”. In this case, the way the person is classified has changed, but this is due to the fact that the biological characteristics of the person have changed. This is still compatible with the category being a natural kind. Although being classified as “obese” results in the person trying to alter how he or she is classified, the category “obese” is still taken to indicate a set of properties that tend to be held together by causal processes.

The above indicates that interactive kinds that are dependent on people’s attitudes in the weak sense can still be homeostatic property clusters. Indeed, Kuorikoski and Pöyhönen (2012) suggest that some interactive kinds are best understood as homeostatic property clusters where the homeostatic causal mechanisms sustaining the clusters comprise both biological and social processes. Under this view, the interactive kind “obesity” can be analyzed as a homeostatic property cluster whose homeostatic causal mechanisms include various metabolic processes, as well as the stabilizing and destabilizing effects of people’s attitudes and other social factors on these metabolic processes. Although the category is dynamic, the various properties in the cluster are causally related.

The ways in which age categories are dependent on people’s attitudes appear to involve both strong and weak senses. To certain extents, the boundaries between some age categories have changed because the norms and conventions that inform judgments about how “young” people and “old” people are expected to behave have changed. Nonetheless, in some cases, the shifts in these boundaries are not merely conventional, but are due to changes across history in the biological, psychological, and social properties of people relative to their chronological ages. Therefore, the ways in which age categories are dependent on people’s attitudes could still be consistent with some age categories being homeostatic property clusters. As we shall see, however, whether or not they can be considered to be natural kinds will also depend on which properties in the clusters are prioritized by the inferential practices of different disciplines.

Hacking (1992) suggests another reason why people’s caring about how they are classified precludes the categories from being natural kinds. This is that the looping effects that occur when people care about how they are classified take place at much faster rates than the feedback effects that influence kinds such as corn and marijuana. The worry, then, is that the fast rates at which the characteristics of interactive kinds change confounds our attempts to use them to support inductive inferences (Cooper, 2004). As noted above, the characteristics of corn and marijuana do change over time, but the rates at which they change are slow enough for the categories to be sufficiently stable epistemic resources. Accordingly, Ron Mallon notes that in these sorts of cases we may still “have knowledge of members of these changing kinds that allows us to engage in successful induction, prediction, explanation, and intervention because our capacity to gain accurate knowledge of these kinds can (sometimes) be far more rapid than the processes that underwrite biological change” (Mallon, 2016, p. 166). By contrast, if the rates at which people alter how they are classified are fast enough to compound the abilities of the categories to support inductive inferences, then a case could be made that they are insufficiently stable to be considered natural kinds. Hence, as Khalidi notes, “after successive iterations of the looping effect, it seems that we may no longer be dealing with the same thing we started with” (Khalidi, 2010, p. 342).

Jessica Laimann raises a related concern, which is that certain interactive kinds are capricious. Their members do not only change at fast rates, but also “behave in wayward, unexpected manners that defeats existing theoretical understanding” (Laimann, 2020, p. 1043). Under Laimann’s account, this sort of interactive kind is a hybrid concept involving a base kind and an associated status kind. For example, the category “unemployed” has a base kind that is defined by a social institution as “being without paid work but available to work”, but is also associated with a status kind that “among other things, involves social stigma” (Laimann, 2020, p. 1061). The ways in which the base properties and the status properties causally interact with one another may change over time and vary across different contexts, which can confound attempts to predict how members of the category will behave in different circumstances. For example, people who are classified as “unemployed” elicit different social attitudes and are likely to behave in different ways depending on whether they reside in a society with a strong welfare system or in a society with a strict austerity program, which in turn may lead to differences in how the base kind and the status kind interact and modify each other.

If age categories turn out to be capricious or too dynamic, then the properties they are supposed to capture may not cluster together tightly enough to yield reliable inductive inferences. Accordingly, they may not be to be considered natural kinds. However, whether or not age categories actually are capricious cannot be determined a priori, but must be assessed relative to the particular interests and purposes of the different disciplines that employ them. Given that different disciplines prioritize different properties in the clusters that make up age categories, which categories turn out to be useful may vary across disciplines. Below, I consider the different ways in which the looping effects of age categories affect epistemic practices in public health, medicine, and politics.

The different interests of public health and medicine can be illustrated with the example of the chronologically defined age category “older adulthood”. Public health is predominantly concerned with improving health at a population level through a variety of interventions, including screening programs, educational campaigns to change health behaviors, and social policies to address health inequalities. Accordingly, it is interested in diverse biological, psychological, and social risk factors, and employs age categories as rough indicators of some of these risk factors that tend to cluster together. The category of “older adulthood” is of interest to public health because its members tend to possess certain properties that are associated with greater healthcare requirements (Walker 2017). However, as noted above, looping effects are resulting in people staying biologically, psychologically, and socially younger for longer relative to their chronological age, and so the category of “older adulthood” is changing.

In spite of this, the chronologically defined age category “older adulthood” may still be epistemically useful in public health, as it indicates broad yet statistically significant differences between the members of this category and the members of other age categories. However, in order to account for the changing characteristics of its members, the chronological age range that is used to define the category must be regularly revised. Indeed, this is recognized by public health practitioners and policy makers. We saw earlier that the Joint Committee of Japan Gerontological Society and the Japan Geriatrics Society have proposed raising the lower chronological age boundary of “older adulthood” to 75 to account for the ways in which people are remaining younger for longer in Japan (Ouchi et al. 2017). Furthermore, to account for geographical differences in rates of senescence, the World Health Organization (2002) has suggested 50 as the chronological age at which “older adulthood” begins in the parts of Africa most affected by poverty.

While the interventions of public health rely on knowledge of broad differences between sections of the population, the targeted interventions of medicine rely on knowledge of the biological properties of individuals. Accordingly, insofar as medicine is concerned with age, it prioritizes the properties associated with biological age. Here, chronologically defined age categories may not be optimal for capturing these properties. As noted earlier, rates of senescence are affected by looping effects and other contingencies, which result in considerable heterogeneity in the biological properties of the members of a given chronological age category, such as “older adulthood” (Nathan, 2021). Given that medical interventions often rely on more detailed knowledge of biological differences between individuals, using a different classification method may be preferable to using a chronologically defined age category.

A possible alternative approach to age classification is to use a more direct measure, or biomarker, of biological age that does not rely on chronological age (Green and Hillersdal, 2021). Magda Hamczyk and colleagues suggest using such a measure for the medical purpose of preventing cardiovascular disease (Hamczyk et al., 2020). Because the members of a given chronological age category vary greatly with respect to their physiological capacities, such a chronological age category may not serve as a useful guide for preventative measures. Instead, by measuring certain biological markers, people can be classified according to their biological ages, which in turn could inform more targeted interventions. However, this approach involves various medical tests and laboratory investigations, which make it too impractical and costly to implement at a population level. Therefore, while it may be helpful for the purpose of medicine, relying on biological age would be too impractical for the purpose of public health.

Politics tends to prioritize the properties associated with social age. Chronological age categories are often used in political analysis on the assumption that they indicate certain behavioral trends and social roles. For example, in the 2017 general election in the United Kingdom, it was widely conjectured that the strong performance of the Labour Party was due to a high turnout rate among “young people” aged 18 to 24. This conjecture was partly informed by evidence that voting patterns vary with age because of changing roles and increasing resources as people get older (Strate et al., 1989). However, this conjecture turned out to be incorrect. An analysis by Christopher Prosser and colleagues found no evidence of a substantially increased turnout rate among people aged 18 to 24 (Prosser et al., 2020). Instead, the strong performance of the Labour Party was attributed to the high turnout rate among people aged 25 to 44, especially those aged 35 to 44 (Bush, 2018). This was partly attributed to the effects of changing political and cultural circumstances on the social roles people typically perform at given chronological ages. These included people having children later and remaining in rented accommodation for longer, which led to people aged 35 to 44 having more concerns about funding cuts to schools and rental properties. Accordingly, the commentator Stephen Bush (2018) has proposed that “young people” should include people in this age range.

Given that the properties associated with social age are highly sensitive to the looping effects of changing political and cultural circumstances, they may be too capricious to be adequately captured by chronological age properties for the purpose of political analysis. The social roles and behaviors of people currently aged 35 to 44 may be very different from the social roles and behaviors of people who were aged 35 to 44 a generation ago. Thus, inferences that are based on chronological age categories may not be straightforwardly generalizable across generations. In light of this, a possible alternative approach to classification may be to use generational categories instead of chronological age categories. Roughly speaking, the people in the generational category “generation Z” are those who presently fall into the chronological age category “adolescence”, while the people in the generational category “millennials” are those who presently fall into the chronological age category “young adulthood”. However, a generational category such as “millennials” takes a more idiographic approach that treats a given generation as a *sui generis* study population influenced by its own set of contingent historical and cultural developments. This approach not only acknowledges that a person of a given chronological age from the present generation is likely to differ from a person from a past generation at that same chronological age, but it also draws attention to longitudinal information about the people classified in that generational category, such as the historical, cultural, political, and technological events that influenced how they developed. These can be useful for explaining why people who currently fall within a certain age range tend to exhibit certain behavioral patterns without assuming that these behavioral patterns generalize across generations.

As well as being helpful for political analysis, generational categories may occasionally be helpful for public health, especially where the health outcome of interest is heavily influenced by the changing cultural context. For example, in previous decades, “adolescence” and “young adulthood” were associated with relatively low rates of suicide, with the highest rates being associated with the category “middle adulthood”. However, partly due to the effects of various social and economic changes, including austerity, political disenchantment, and the climate crisis, rates of mental ill health and suicide have significantly increased among the current generation of people classified in “young adulthood” (Khazan, 2020). Hence, in light of these changing social and economic circumstances, we may struggle to make robust inferences about the patterns of mental ill health and suicide among “young people” in the current generation based on the patterns of mental ill health and suicide among “young people” from the previous generation. Rather, generational categories may be more helpful, insofar as they could facilitate more idiographic examinations of the different social and economic circumstances endured by different generational cohorts, as well as how these influenced the patterns of mental ill health and suicide in these generational cohorts.

To summarize, we have seen that the extents to which looping effects confound our abilities to use chronologically defined age categories to guide inductive inferences vary depending on the particular categories that are used, the purposes of different disciplines, and the properties that are prioritized by these disciplines. Some categories may be useful in public health, although the chronological age boundaries may need to be revised regularly to account for the changing characteristics of their members. However, in medicine and politics, which prioritize different properties and have different epistemic requirements, different classificatory approaches may be more optimal. This lends support to a pluralistic approach to natural kindhood, such as John Dupré’s (1993) promiscuous realism, according to which there are many defensible ways of classifying individuals “and the best way of doing so will depend on both the purposes of the classification and the peculiarities of the organisms in question, whether those purposes belong to what is traditionally considered part of science or part of ordinary life” (Dupré, 1993, p. 57). Indeed, this is also implicated by Boyd’s (1999a, 1999b) homeostatic property cluster account, which acknowledges that natural kindhood is discipline relative. Thus, the mere fact that an age category is an interactive kind is insufficient to preclude its being a natural kind. Rather, this is informed by whether or not the looping effects compromise the inferential practices of different disciplines.

## Conclusion

The ontological status of age classification presents a philosophical challenge with implications for practice and policy. Chronologically defined age categories are supposed to capture certain biological, psychological, and social properties of people, which can be used to inform inductive inferences in various disciplines, including public health, medicine, and politics. Given that these properties tend to cluster together due to causal processes, age categories *prima facie* seem to qualify as natural kinds under a homeostatic property cluster account of natural kindhood. However, I have shown that age categories are also affected by dynamics that make them historically unstable. First, due to healthcare and cultural developments, the biological, psychological, and social characteristics of people are changing across history relative to their chronological ages, such that people are staying younger for longer. Second, given the normative connotations associated with age, the practice of classifying people into age categories motivates them to change their characteristics in order to alter how they are classified. Accordingly, age categories are best understood as interactive kinds that are influenced to varying degrees by social and biological looping effects. Social feedback occurs when the valorization of youth in society prompts people to modify their behaviors and social roles, such that they are staying socially younger for longer. Biological feedback occurs when people respond to how they are classified by modifying their health behaviors in ways that delay senescence and allow them to stay biologically younger for longer.

The fact that age categories are interactive kinds does not, on its own, preclude them from being considered natural kinds. In some cases, interactive kinds can be understood as homeostatic property clusters, insofar as the biological and social looping effects can be included among the homeostatic causal mechanisms that connect the properties. However, in cases where these looping effects result in people’s characteristics changing across history at fast rates or in wayward manners, the properties may not cluster together tightly enough to support our attempts to use the categories in inductive inferences. In these cases, the categories may be too unstable or capricious to be considered natural kinds, at least under an epistemological approach to natural kindhood that takes natural kinds to be categories that support our inferential practices. Given that the inferential practices of different disciplines prioritize different properties and have different purposes, whether or not age categories are too unstable or capricious can only be assessed relative to particular disciplines. I have suggested that at for at least some purposes, some chronologically defined age categories may be too unstable to yield sufficiently reliable inductive inferences. This provides a reason for us to revise the classificatory practices of some disciplines, which may include reviewing the chronological age boundaries through which the categories are defined and, in some cases, relying on measures other than chronological age.
